# Longitudinal characterization of TK6 cells sequentially adapted to animal product-free, chemically defined culture medium: considerations for genotoxicity studies

**DOI:** 10.3389/ftox.2023.1177586

**Published:** 2023-07-04

**Authors:** Noelia Perez-Diaz, Ewelina Hoffman, Julie Clements, Rebecca Cruickshank, Ann Doherty, Daniel Ebner, Joanne Elloway, Jianan Fu, Joanne Kelsall, Val Millar, Ouarda Saib, Andrew Scott, Ian Woods, Victoria Hutter

**Affiliations:** ^1^ ImmuONE Limited, Hatfield, United Kingdom; ^2^ LabCorp Drug Development, Harrogate, United Kingdom; ^3^ PAN-Biotech UK Ltd., Wimborne, United Kingdom; ^4^ Safety Sciences, Clinical Pharmacology and Safety Sciences R&D, AstraZeneca, Cambridge, United Kingdom; ^5^ Nuffield Department of Medicine, Target Discovery Institute, University of Oxford, Oxford, United Kingdom; ^6^ PAN-Biotech GmbH, Aidenbach, Germany; ^7^ Safety and Environmental Assurance Centre (SEAC), Unilever, Bedford, United Kingdom; ^8^ LabCorp Drug Development, Huntington, United Kingdom; ^9^ Centre for Topical Drug Delivery and Toxicology School of Life and Medical Sciences, University of Hertfordshire, Hatfield, United Kingdom

**Keywords:** chemically defined serum replacement, fetal bovine serum, FBS replacement, sequential adaptation, TK6, OECD, serum-free, TG 487

## Abstract

**Introduction:** *In vitro* approaches are an essential tool in screening for toxicity of new chemicals, products and therapeutics. To increase the reproducibility and human relevance of these *in vitro* assessments, it is advocated to remove animal-derived products such as foetal bovine serum (FBS) from the cell culture system. Currently, FBS is routinely used as a supplement in cell culture medium, but batch-to-batch variability may introduce inconsistency in inter- and intra-lab assessments. Several chemically defined serum replacements (CDSR) have been developed to provide an alternative to FBS, but not every cell line adapts easily and successfully to CDSR-supplemented medium, and the long-term effect on cell characteristics remains uncertain.

**Aim:** The aim of this study was to adapt the TK6 cell line to animal-product free CDSR-supplemented medium and evaluate the long-term effects on cell health, growth, morphology, phenotype, and function. This included a provisional assessment to determine the suitability of the transitioned cell line for standardised genotoxicity testing using the “*in vitro* mammalian cell micronucleus test” (OECD TG 487).

**Materials and methods:** Gradual adaptation and direct adaptation methodologies were compared by assessing the cell proliferation, size and viability every passage until the cells were fully adapted to animal-free CDSR. The metabolic activity and membrane integrity was assessed every 4-8 passages by PrestoBlue and CytoTox-ONE™ Homogeneous Membrane Integrity Assay respectively. A detailed morphology study by high content imaging was performed and the expression of cell surface markers (CD19 and CD20) was conducted via flow cytometry to assess the potential for phenotypic drift during longer term culture of TK6 in animal-free conditions. Finally, functionality of cells in the OECD TG 487 assay was evaluated.

**Results:** The baseline characteristics of TK6 cells cultured in FBS-supplemented medium were established and variability among passages was used to set up acceptance criteria for CDSR adapted cells. TK6 were adapted to CDSR supplemented medium either via direct or gradual transition reducing from 10% v/v FBS to 0% v/v FBS. The cell growth rate was compromised in the direct adaptation and therefore the gradual adaptation was preferred to investigate the long-term effects of animal-free CDSR on TK6 cells. The new animal cells showed comparable (*p* > 0.05) viability and cell size as the parent FBS-supplemented cells, with the exception of growth rate. The new animal free cells showed a lag phase double the length of the original cells. Cell morphology (cellular and nuclear area, sphericity) and phenotype (CD19 and CD20 surface markers) were in line (*p* > 0.05) with the original cells. The new cells cultured in CDSR-supplemented medium performed satisfactory in a pilot OECD TG 487 assay with compounds not requiring metabolic activation.

**Conclusion:** TK6 cells were successfully transitioned to FBS- and animal product-free medium. The new cell cultures were viable and mimicked the characteristics of FBS-cultured cells. The gradual transition methodology utilised in this study can also be applied to other cell lines of interest. Maintaining cells in CDSR-supplemented medium eliminates variability from FBS, which in turn is likely to increase the reproducibility of *in vitro* experiments. Furthermore, removal of animal derived products from cell culture techniques is likely to increase the human relevance of *in vitro* methodologies.

## 1 Introduction

*In vivo* animal models are widely utilised for both research and industrial toxicity programmes, however there are vast differences in animal (mouse, rat, dog) physiology, biochemistry and pharmacology which questions their relevance in understanding the safety of substances in humans (Knudsen and Ochs, 2018). There are an increasing number of human *in vitro* cell culture systems and assessment strategies within toxicology and a growing number of these are being included in the Organization for Economic Co-operation and Development (OECD) Guidelines for the Testing of Chemicals and becoming the international standard for assessing the potential effects of chemicals on human health (European Commission, Joint Research Centre 2019). However, animal-derived products (proteins, enzymes, antibodies) are still widely relied upon for culturing human cells and are required as part of the assessment methodology. There are increasing pressures for these *in vitro* assays to also be free from animal-derived products in order to improve human relevance and reproducibility and reduce the use of animals (Gstraunthaler, Lindl and van der Valk, 2013).

One of the most widely used animal products in cell culture is animal-derived serum, in particular fetal bovine serum (FBS) which is added to cell culture medium to support cell adhesion and proliferation processes (Hayman et al., 1985). FBS is harvested from the blood of a bovine fetus after removal from the slaughtered cow standardly by cardiac puncture, which raises ethical concerns due to the potential suffering of the foetus (J van der Valk et al., 2004; van der Valk et al., 2018). FBS supplementation creates substantial risks to the cells growing *in vitro*, for example, the potential introduction of pathogens such as endotoxins, *mycoplasma*, virus and prions into the cell culture (Kozasa et al., 2011). Furthermore, batch-to-batch FBS variability can cause uncharacterised interactions with test substances and modifications to the behaviour of the cells, resulting in non-reproducible data and requires costly and time-consuming batch validation to assess its reproducibility (J. van der Valk et al., 2004). The presence of animal-derived serum has also been shown to alter the biological responses of some human cell types, which questions the relevance of data generated (Shahdadfar et al., 2005). Jacobs et al. (2019) proposed the substitution of FBS for human serum, and two cell lines have been successfully validated in two different OECD guidelines using this approach, KeratinoSensTM (OECD TG 442 days) (Belot et al., 2017) and h-CLAT (OECD TG 442e) (Edwards et al., 2018). Although human serum solves the issue of the usage of animal-derived products, the procedure still has the problem of batch-to-batch variability from human donors, ethical concerns and availability as well as the potential contamination of the cultures from pathogens (Jacobs et al., 2019b).

It is becoming increasingly acknowledged that to attain improved experimental reproducibility, all constituents of the cell culture medium and their influence on cellular process should be established (Baker, 2016; Hirsch and Schildknecht, 2019). A number of defined serum replacements have been developed to provide an alternative to human and animal sera. These defined serum replacements typically contain purified proteins, lipids, salts, amino acids, trace elements, hormones and may also contain growth factors and undefined hydrolysates and peptones (van der Valk et al., 2010). Whilst serum replacements avoid the issues of batch-to-batch variability and introduction of pathogens, the highly purified growth factors, hormones and proteins incorporated are typically from animal origin making the replacement not animal-free (Marigliani, Silva, Balottin, da Silva, et al., 2019). In contrast chemically defined serum replacements (CDSR) contain only materials that are molecularly homogenous or mixtures of characterised and quantified ingredients (Usta et al., 2014). However, the main limitation of CDSR is that not every cell line adapts easily and successfully (Marigliani, Silva, Balottin, Silva, et al., 2019). Sometimes the formulation can be cell-line specific, and it can be very technically challenging to ensure the medium contains all the required components for a cell type (van der Valk et al., 2010; 2018). Furthermore, the long-term effects on the cell line also remain uncertain (Marigliani, Balottin and Augusto, 2019). For these reasons the uptake of CDSR in human cell culture remain slow despite many available products being commercially available.

In this context, our aim was to characterise the effect of CDSR on morphological, viability and phenotypical cell characteristics in comparison with standard FBS-containing culture medium. The purpose of this study was to understand the influence of CDSR medium and transition methodology (gradual adaptation vs. direct adaptation) to evaluate the potential for these animal-free cell line in globally standardised *in vitro* assessments. The human lymphoblast TK6 cell line was used as it is associated with a number of OECD test guidelines (OECD TG 473, OECD TG 476, OECD TG 487, OECD TG 490). Baseline characteristics were established for TK6 cultured in standard FBS-containing medium to understand the variability of cells cultured in the standard protocol. The effect of both direct and gradual adaptation methods of transitioning cells to were investigated and longitudinal characterisation conducted over 48 sub-cultures. A variety of tools including average population health and morphology assessments, proliferation rates, protein expression and high content image analysis to assess morphological changes within individual cells was applied to develop a thorough understanding of how the TK6 cell line responds to CDSR in the short and longer term in culture.

## 2 Materials and methods

### 2.1 Cell culture and cell transition

TK6 cells (P+2) were purchased from Merck (Merck, United Kingdom) and cultured in RPMI 1640 GlutaMAX^TM^ medium (ThermoFisher, United Kingdom) containing 2 mM L-Glutamine (Sigma, United Kingdom), 1 mM sodium pyruvate (Sigma, United Kingdom), 10 mM HEPES (Sigma, United Kingdom), 1% v/v penicillin/streptomycin (Merck, United Kingdom), and 10% v/v FBS (FBS Supreme, PAN-Biotech, United Kingdom). Cells were cultured in 25 cm^2^ T-flasks, passaged every 3 or 4 days at cell density 1 × 10^5^ cells/mL. FBS was sequentially reduced from 10% v/v during sub-culture when cell growth and viability indicated they were adapted to the new condition, until FBS was completely substituted for the chemically defined animal-free serum replacement, Panexin CD (PAN-Biotech, United Kingdom). Cells were kept in all FBS concentrations for long-term characterization until the end of the study. Additionally, cells growing in 10% v/v FBS were directly transferred to 0% v/v FBS (10% v/v CDSR) and monitored for cell growth, viability, and cell diameter for the following 18 passages.

### 2.2 Cell viability, size and growth assessment

Cell growth, viability and cell diameter were monitored in every passage using the Countess 3 Automated Cell Counter (ThermoFisher, United Kingdom). Data collected every 3 passages was pooled for statistical analysis and early detection of CDSR effects on the characteristics assessed.

### 2.3 Growth kinetics

The growth kinetics of TK6 cells cultured in 10% v/v FBS- or 10% v/v CDSR-supplemented medium were calculated. Briefly, 10,000 cells per well (in 100 µL) were seeded in triplicates per time point in a 96 well plate and manually counted at 24, 48, 72, 96, and 168 h. Cell counts were plotted against the number of hours cultured, and the slope of the linear phase of the exponential growth was used to determine the population doubling time.

### 2.4 Cell metabolic activity and cytotoxicity

The PrestoBlue™ cell viability reagent (Invitrogen, United Kingdom) was used to assess cell metabolic activity according to manufacturer’s instructions. A 10-fold dilution of PrestoBlue™ reagent was added to samples, which were then incubated for 1 h at 37°C, in the dark. Following incubation, 50 µL of samples were transferred to a black 96-well plate and fluorescence measurements (Ex/Em: 525/590) were recorded using CLARIOstar^®^ plate reader (BMG Labtech, United Kingdom).

The CytoTox-ONE™ Homogeneous Membrane Integrity Assay kit (Promega, United Kingdom) was used to assess the membrane integrity according to manufacturer’s instructions. The selected samples were lysed with 10% v/v Triton-X to serve as positive control and equal volumes (50 µL) of CytoTox-ONE™ reagent were added to cell culture supernatant, cell lysates and blanks. Following a 10-min incubation at room temperature, in the dark, the reaction was stopped using 50 µL of the Stop Solution. Fluorescence measurements (Ex/Em: 560/590) were recorded using CLARIOstar^®^ plate reader (BMG Labtech, United Kingdom).

### 2.5 Cell morphology

The morphology of TK6 cells was assessed during gradual adaptation and long-term incubation in CDSR-supplemented medium by high content imaging, as described previously (Hoffman et al., 2017). In brief, cells were stained with 10 μg/mL Hoechst 33342 (Invitrogen, United Kingdom) for 30 min. Cells were washed once with 100 μL PBS and fixed with 3.7% w/v paraformaldehyde in the dark for 15 min. Fixed cells were stained overnight with Cell Mask Deep Red (Invitrogen, United Kingdom) diluted 1:1,000 (according to the manufacturer’s protocol) and washed once with PBS before imaging. Samples were stored at 4°C in the dark for up to 7 days prior to sample acquisition. Images were captured using the IN Cell Analyser 6000 (Cytiva, United Kingdom) with a ×40 objective in standard 2D imaging mode with an exposure time of 0.1 s. Image analysis was performed using In Cell Developer Toolbox v 1.9.2, Level 3 analysis (Cytiva, United Kingdom). In brief, the cell nuclear dye Hoechst 33342 was used to identify nucleated cells while Cell Mask Deep Red staining highlighted cell cytoplasm. Each sample was imaged using 12 fields capturing in total between 500 and 1,500 cells per well. Quantitative measurements for each cell were generated from the image analysis for cellular area, nuclear area, and cell sphericity.

### 2.6 Cell phenotype

TK6 cells are CD19 positive and 50% of the population is CD20 positive according to the cell line description (ECACC Culture Collections, United Kingdom). To test any effect of CDSR on TK6 phenotype, the expression of CD19 and CD20 was measured by flow cytometry. Briefly, aliquots of 5 × 10^5^ cells were blocked with 10% v/v human serum in staining buffer (2% v/v FBS in PBS) for 15 min at 4°C. Cells were washed once with staining buffer and incubated in staining solution containing fluorochrome-labelled monoclonal antibodies anti-human CD19 or anti-human CD20 (Invitrogen, United Kingdom) or the relevant isotypes Mouse IgG1 Isotype CD19 and Mouse IgG2 Isotype CD20 (Invitrogen, United Kingdom) for 45 min at 4°C in the dark. Following incubation, cells were washed once with staining buffer, once with PBS and finally fixed with 3.7% w/v paraformaldehyde solution. After 15 min fixation at room temperature in the dark, cells were washed with PBS and aliquoted in triplicates in a 96-well plate. An unstained sample and the appropriate isotype controls were included in each analysis to address autofluorescence and non-specific binding, respectively. Flow cytometric measurements were performed using Guava EasyCyte 8HT (Millipore, United Kingdom). Each sample population was gated to only include cells of interest based on their forward scatter (cell size) and/or side scatter (cell granularity) profiles. A total of 10,000 events were collected per sample. Raw data was analysed using GuavaSoft 3.1.1 software (Millipore, United Kingdom).

### 2.7 Micronucleus assay

TK6 were seeded on 96 well plates at 3 × 10^4^ cells per well and incubated at 37°C for either 24 h (TK6 cultured in 10% v/v FBS supplemented medium) or 48 h (TK6 cultured in 0% v/v FBS supplemented medium). Then, cells were treated with 0.05 and 0.15 μg/mL mitomycin C (Merck, United Kingdom) or 0.03 and 0.09 μg/mL colchicine (Merck, United Kingdom) and incubated for 3 h at standard cell culture conditions. Subsequently, the treatments were removed, replaced with fresh drug-free medium and cells followed 24 h recovery period. For micronucleus staining, at the end of the recovery period plates were centrifuged at ×200 g for 5 min at room temperature. In order to prevent cells from being removed due to aspiration, approximately half of the media in the plate was removed and discarded. A solution of staining fixative containing Hoechst (Invitrogen, United Kingdom) in 3.7% paraformaldehyde (ThermoFisher, United Kingdom) was made. 100 μL of the staining solution was added, then 100 µL was removed and discarded. This adding and removing step was performed 3 times. The plates were left for 30 min in the dark at room temperature. 100 μL of the staining solution was removed and 100 µL of PBS was added to each well using the same method as the staining solution. Cells were visualised using a fluorescent microscope with ×40 magnification (EVOS ThermoFisher, United Kingdom). Mononucleated cells with intact nuclear and cytoplasmic membrane were considered suitable for micronuclei (MN) counting. The parameters for MN scoring followed OECD TG 487 specifications. The MN frequency was obtained by manually assessing 100 mononucleated cells per replicate.

### 2.8 Statistical analysis

A one-way ANOVA analysis with Bonferroni’s multiple comparison *post hoc* tests was used to assess the statistical significance between cells growing in 10% v/v FBS-supplemented medium and cells growing in all the different concentrations of CDSR-supplemented media. Statistical significance was evaluated at a 95% confidence level (*p* < 0.05). All statistical tests were performed using GraphPad Instat^®^ version 9.0.0 (GraphPad Software, San Diego, CA).

## 3 Results

### 3.1 Variability of TK6 cells in FBS culture (baseline characteristics)

**FIGURE 1:**
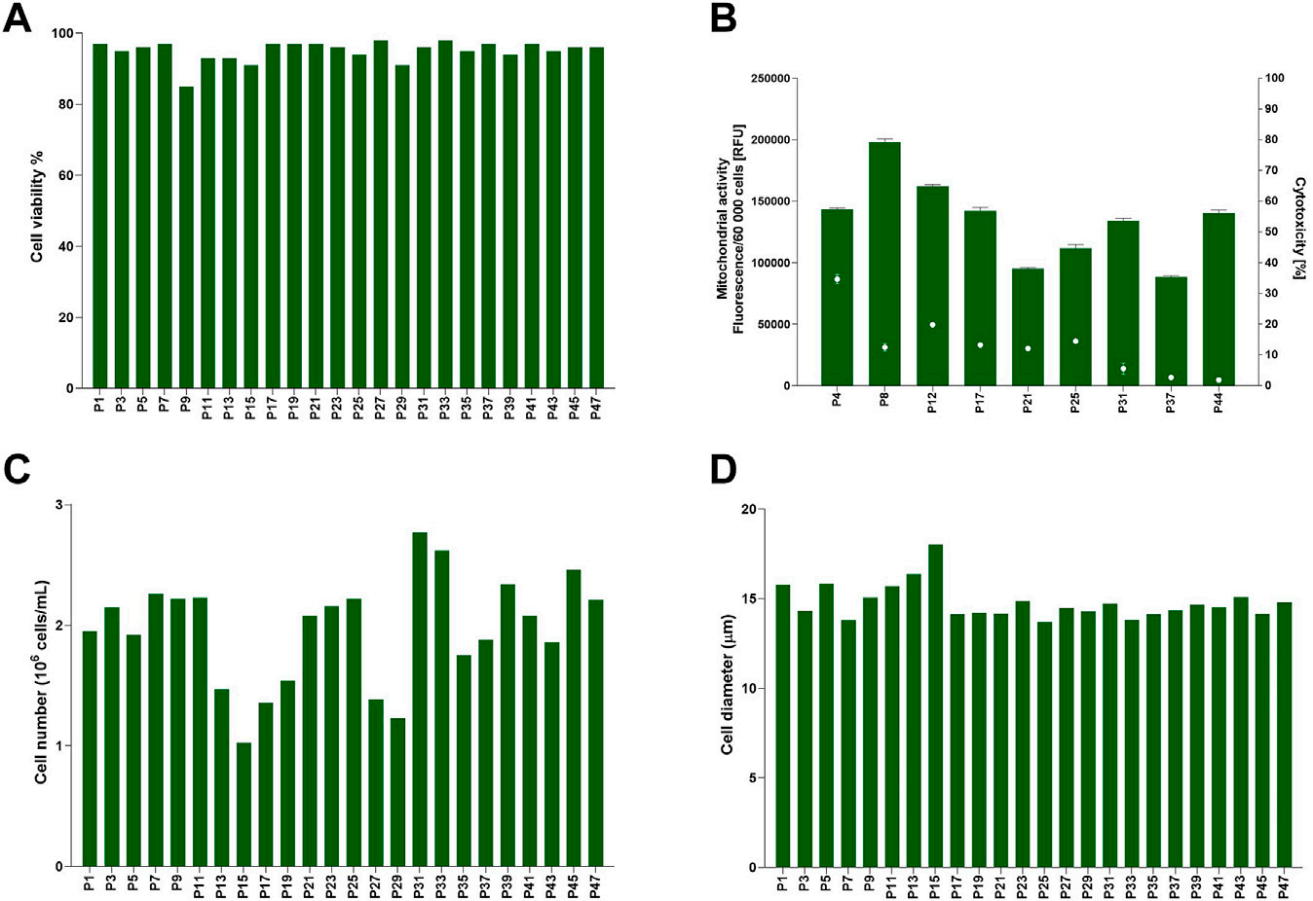
Baseline for TK6 cell line cultured in standard medium composition containing 10% (v/v) FBS. Cell viability **(A)**, cell number **(C)** and cell diameter **(D)** were measured every cell passage. Mitochondrial activity (bars) and cytotoxicity (dots) **(B)** were measured at indicated on *x*-axis passage number and expressed as mean (±SD).

Cell data for TK6 cells cultured in 10% v/v FBS was assessed longitudinally to establish cells characteristics *in vitro* (47 passages, 24 weeks) in culture long-term (Figure 1). Cell viability (Figure 1A) ranged from 85% to 98%. Cell health was additionally confirmed with mitochondrial activity and cytotoxicity assessment (Figure 1B). The maximum toxicity (35%) was reported at passage number 4 (P4), but it decreased to >20% in further sub-cultures, confirming cells were viable and healthy at each passage. Inherent variability was observed for the number of cells counted (cell count) at each passage. Cell number (Figure 1C) varied between 1 × 10^6^ cells/mL to 2.8 × 10^6^ cells/mL with an average 2 (±0.4) × 10^6^ cells/mL. Cell diameter (Figure 1D) was consistently between 13.7 and 18 μm, with an average of 14.8 (±1) µm. This demonstrates the inherent variability of TK6 cell characteristics after sub-culturing in FBS-containing medium with a single batch of FBS. This longitudinal assessment of cell characteristics was used determine acceptance criteria for cells during the CDSR-adaptation process where the maximum and minimum values for cell characteristics were set as acceptance thresholds for the characteristics of CDSR TK6 cultures.

### 3.2 Effect of direct adaptation on cell properties

**FIGURE 2:**
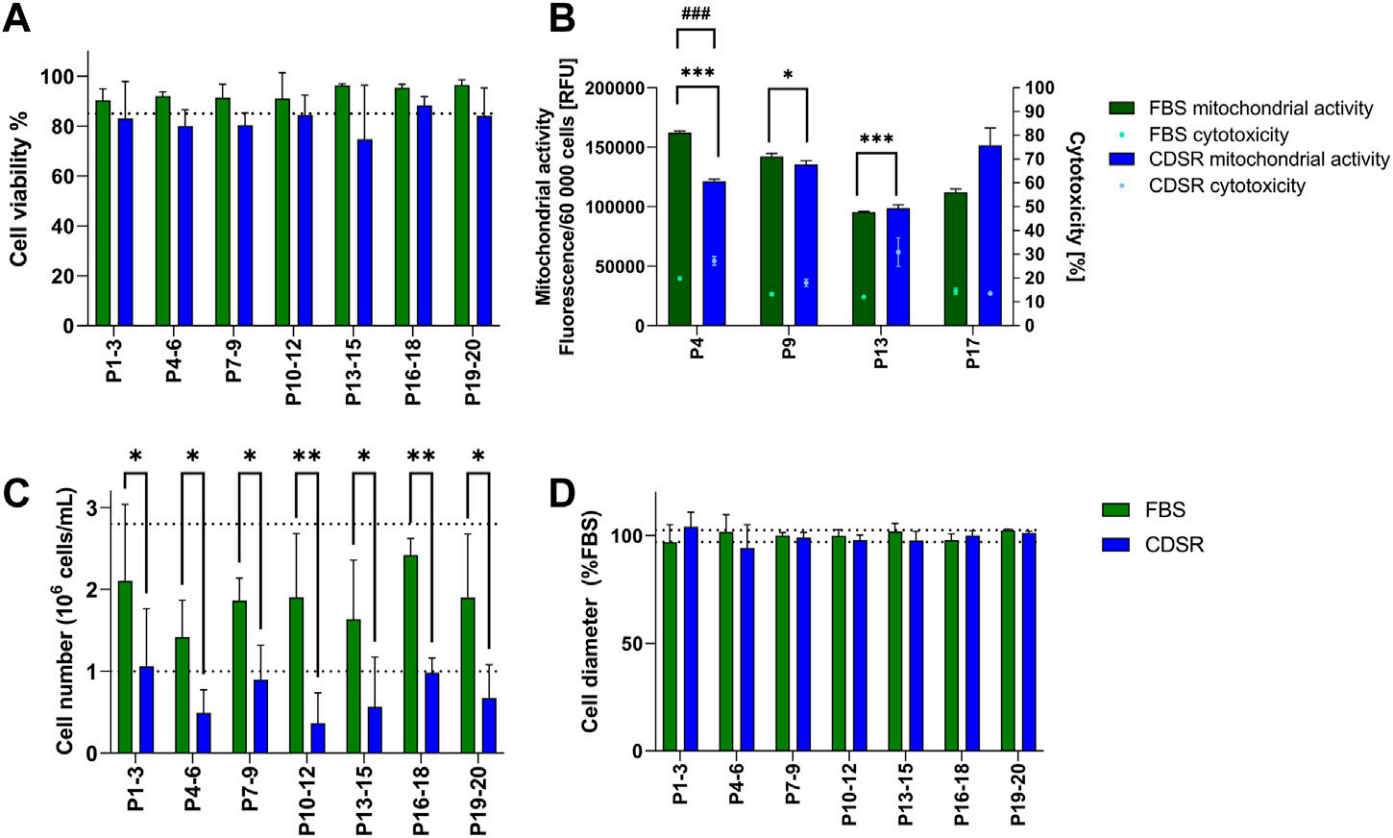
Characterisation of TK6 cell line during direct adaptation to chemically defined serum replacement (CDSR) supplemented media. Cell viability **(A)**, cell count **(C)**, and cell size **(D)** were measured every cell passage and pooled every three passages and expressed as mean (±SD). Mitochondrial activity (bars) and cytotoxicity (dots) **(B)** were measured at indicated on *x*-axis passage number and expressed as mean (±SD). Dotted lines show the highest and/or lowest values of cell viability **(A)**, growth **(C)**, and cell diameter **(D)** of cells growing in 10% (v/v) FBS-supplemented medium. Significant difference between conditions was analysed by two-way ANOVA. Statistical significance was indicated as follows: for **(B)**: cytotoxicity statistical significance was indicated with *: * indicates *p* < 0.05 and *** indicate *p* < 0.001; while mitochondrial activity statistical significance was indicated with #: ### indicate *p* < 0.05; for **(C)**: * indicates *p* < 0.05 and ** indicate *p* < 0.01.

TK6 cells were directly transferred from 10% v/v FBS-supplemented media to 10% v/v CDSR-supplemented media. Subsequently cell proliferation, cell diameter and viability (including mitochondrial activity and cytotoxicity), were monitored for 20 passages (10 weeks) (Figure 2). Viability and cell diameter of TK6 cells in 10% v/v CDSR-supplemented medium were not significantly different (*p* < 0.05) from cells cultured in 10% v/v FBS supplemented medium (Figures 2A, D). However, the viability of TK6 cells directly transferred cells at each passage was continually below the acceptance threshold (Figure 2A). Additionally, cytotoxicity of TK6 cells in 10% v/v CDSR-supplemented medium was significantly lower at passage 4 (*p* < 0.001), passage 9 (*p* < 0.05) and passage 13 (*p* < 0.001) when compared with standard FBS-containing culture, while significantly lower mitochondrial activity was only observed at passage 4 (*p <* 0.05) (Figure 2B). Furthermore, a significant (*p* < 0.05) reduction in cell number (indicating reduced proliferation) was reported at each passage (Figure 2C). Cell proliferation and viability of cells directly adapted to CDSR was below the acceptance threshold and hence this transition methodology was discontinued.

### 3.3 Gradual adaptation

**FIGURE 3:**
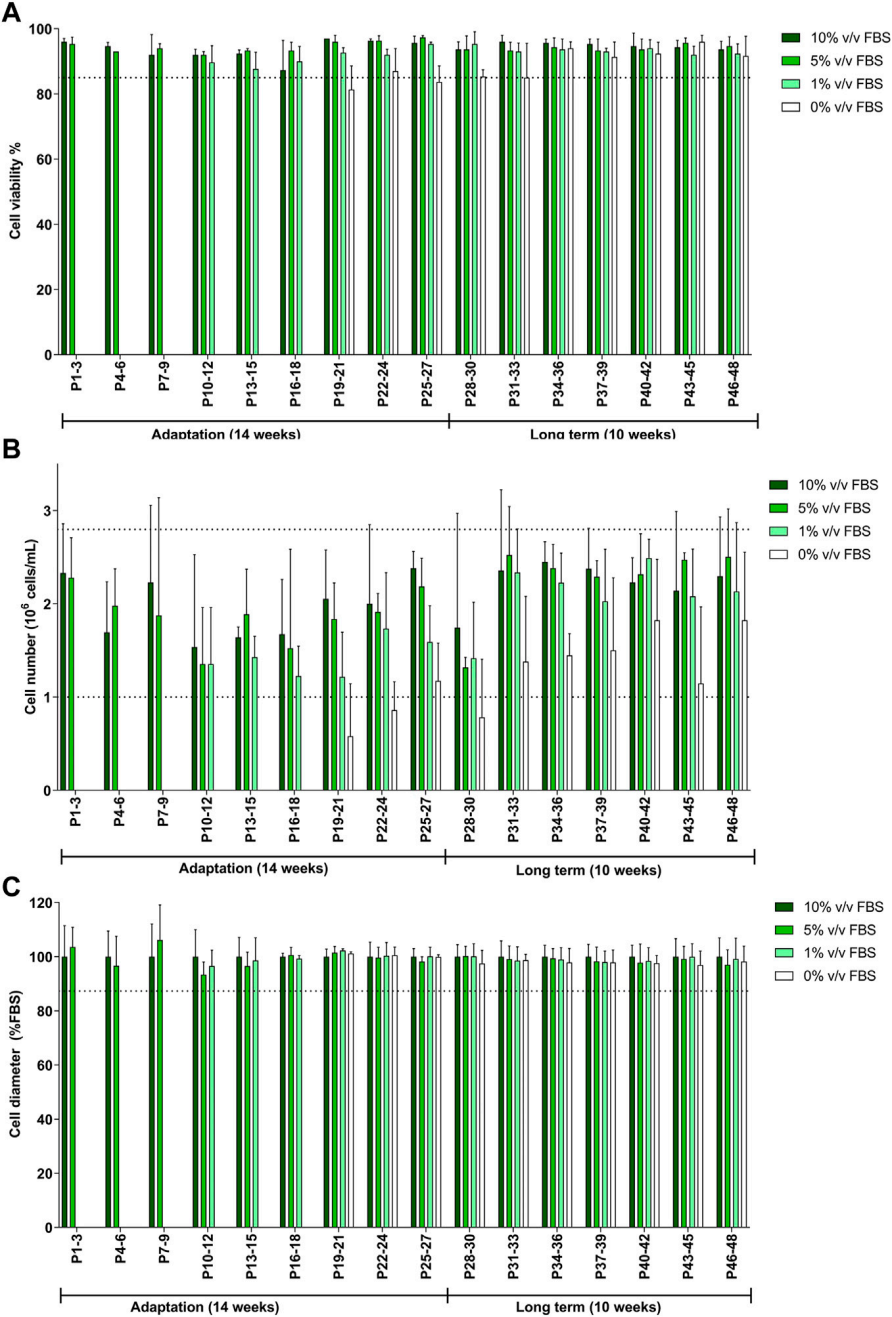
Characterisation of TK6 cell line during gradual adaptation and long term incubation in chemically defined serum replacement (CDSR) supplemented media. Cell viability **(A)**, cell count **(B)**, and cell size **(C)** were measured every cell passage and pooled every three passages and expressed as mean (±SD). Dotted lines show the lowest values of cell viability **(A)**, growth **(C)**, and cell diameter (D) of cells growing in 10% (v/v) FBS-supplemented medium. Significance between conditions was assessed using a one-way ANOVA. The data are presented as mean ± SD.

TK6 cells were gradually transitioned from the 10% v/v FBS-supplemented medium to 10% v/v CDSR-supplemented medium. FBS content was gradually reduced to 5% v/v and 1% v/v until finally substituted for 10% v/v CDSR. Cells were maintained in new medium composition for 8 passages (4 weeks) and observed closely for changes in viability and proliferation before each reduction in FBS. The total length of TK6 adaptation to CDSR-supplemented medium took 27 passages (14 weeks) to be fully adapted to 10% v/v CDSR-supplemented medium (Figure 3). Whilst the cell viability and cell diameter were not affected by the reduction of FBS content and results from passages were above the lower acceptance threshold (Figures 3A, C), the cell number was reduced (below the acceptance criteria) during adaptation process (Figure 3B). However, cell count improved with a time in culture reaching cell number above the exclusion threshold after passage 30, after the adaptation process. After adaptation the animal-product free TK6 cells were maintained for following 10 weeks and showed characteristic (viability, cell number and diameter) within the variability of the original TK6 cells cultured in FBS-containing medium (Figure 3).

### 3.4 Characterisation of animal-product free TK6 cells: growth rate, cell morphology and phenotype

#### 3.4.1 Growth kinetics

**FIGURE 4:**
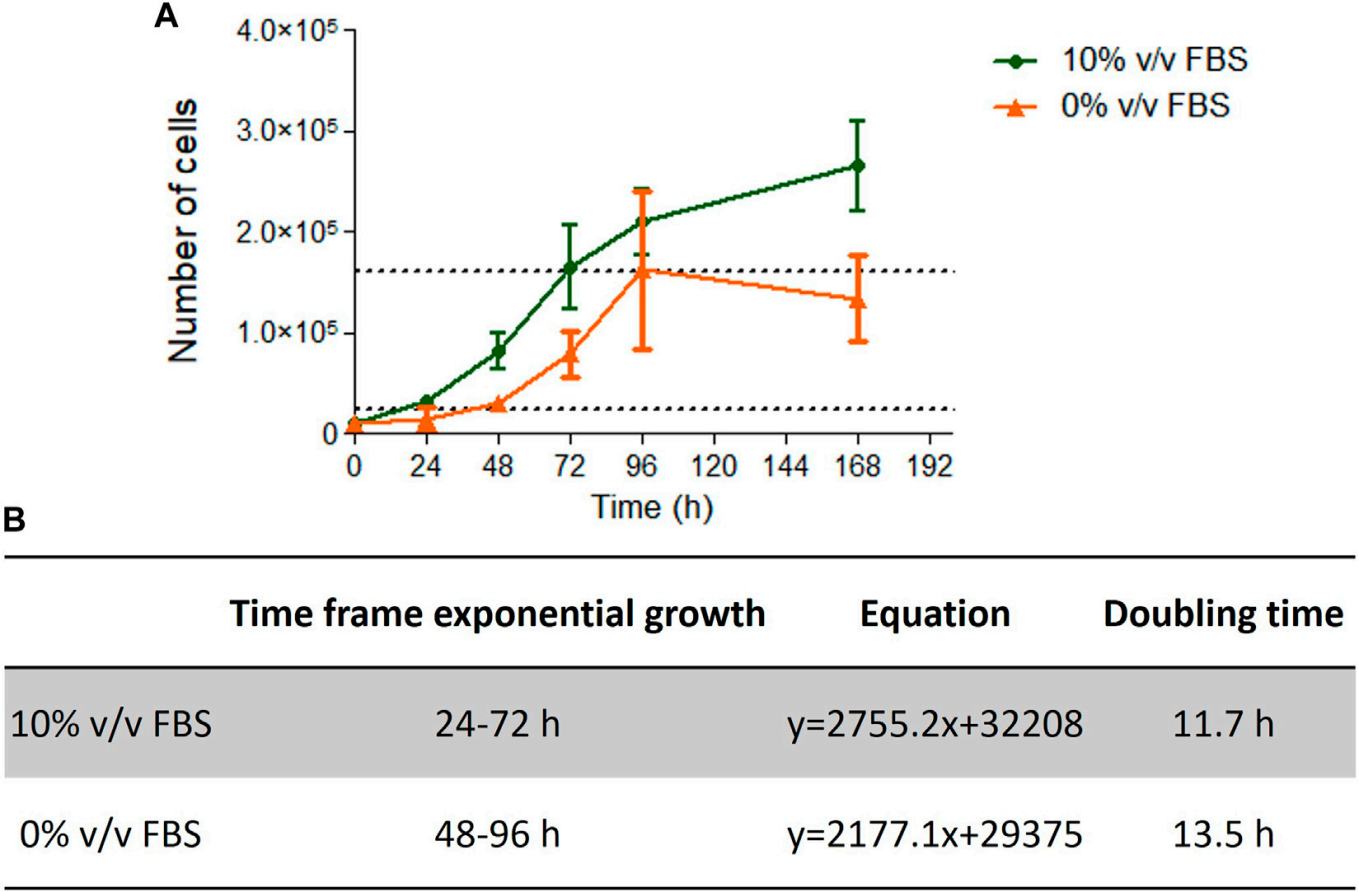
TK6 growth kinetics. Growth curve of TK6 cells growing in FBS- or CDSR-supplemented media **(A)** and details of exponential growth and doubling time calculated from the growth curve **(B)**. Data is the result of *n* = 4 in triplicates and error bars show ±SEM.

Cells cultured in 10% v/v FBS or 10% v/v CDSR demonstrated standard growth kinetic profiles with a 48 h linear exponential growth phase and population doubling times of 11.7 and 13.5 h respectively (Figure 4). These results were with agreement with range of 13–15 h reported for this cell line (Lorge et al., 2016). However, it was noted that cells growing in CDSR-supplemented medium increased the lag phase to 48 h compared with 24 h for cells cultured in FBS-supplemented medium. This extended lag phase should be taken into consideration in experimental design for animal product-free cell lines performing genotoxicity testing.

#### 3.4.2 Cell morphology

**FIGURE 5:**
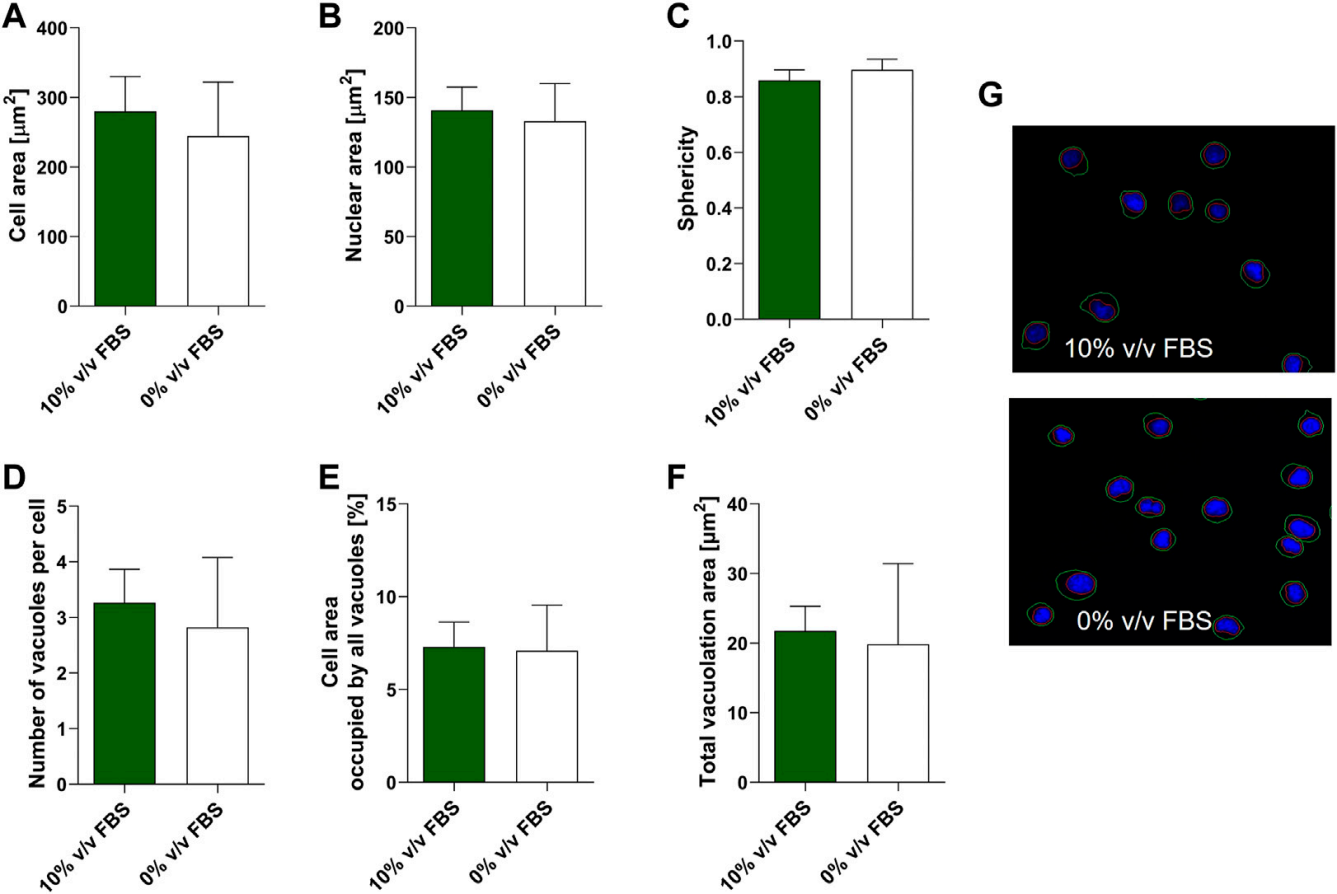
Morphometric characterisation of TK6 cell line cultured in FBS-supplemented medium (green bars) and in CDRS-complemented media (white bars). Cell area **(A)**, nuclear area **(B)**, sphericity **(C)**, number of vacuoles per cell **(D)**, cell area occupied by vacuoles **(E)** and total vacuolation area **(F)** were assessed using high content image analysis. Data are expressed as mean (±SD) of *n* = 3 replicates from two separate experiments on selected passage numbers 35 and 47. Panel **(G)** shows representative images of TK6 cells (×20 magnification) stained with Hoechst 33,342. Nuclear and cellular segmentations are outlined with red (nucleus) and green (cell) colours.

High content image analysis was used to quantify morphological characteristics of TK6 cells including cell area, nuclear area, cell sphericity, number of vacuoles within a cell and the cell area occupied by vacuoles (Figure 5). No significant difference (*p* > 0.05) was observed between TK6 cells cultured in FBS or CDSR supplemented medium for any of the morphometric parameters assessed.

#### 3.4.3 Cell phenotype

**FIGURE 6:**
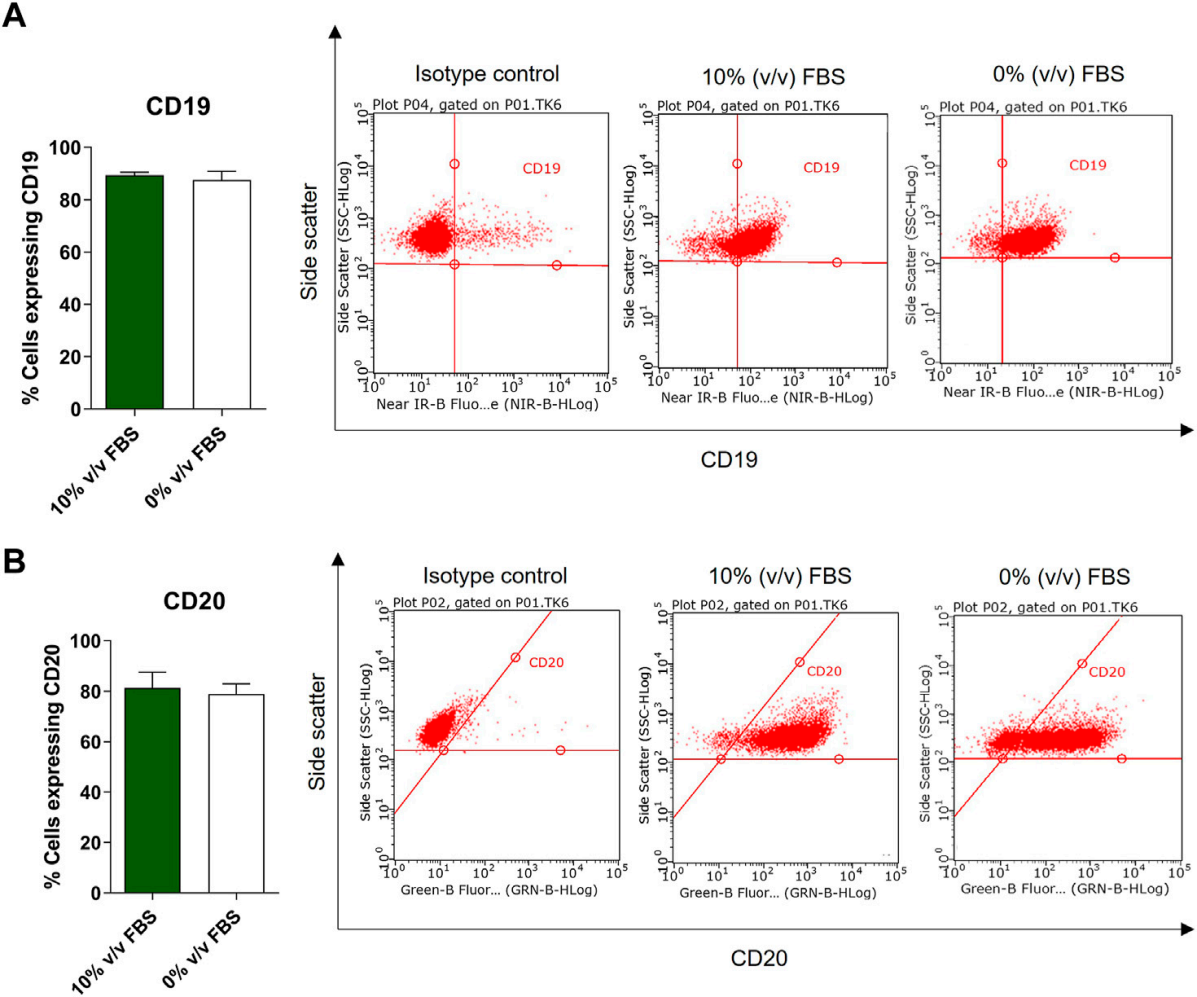
Phenotypic characterisation of TK6 cell line cultured in FBS-supplemented medium (green bars) and in CDRS-complemented media (white bars). CD19 **(A)** and CD20 **(B)** surface markers expression was assessed by flow cytometry. Data are expressed as mean (±SD) of *n* = 3 separate experiments on selected passage numbers 23, 29, and 41.

According to the depositor’s cell line description, TK6 cells are phenotypically characterised as CD19 positive and 50% of the population is CD20 positive (ECACC Culture Collections). Phenotypic analysis was performed on both FBS and CDSR supplemented TK6 cells. No significant difference (*p* > 0.05) in CD19 and CD20 expression was observed between cells cultured in either medium. Both populations expressed CD19 on their surface and over 50% of the cell population expressed the CD20 surface marker confirming the correct phenotype profile for the lymphoblast cell line (Figure 6).

#### 3.4.4 Evaluation of micronuclei induction

To assess suitability of the new animal-product free TK6 cells to genotoxicity testing, the *in vitro* micronucleus assay was performed following OECD TG 487. Both cell lines were evaluated using mitomycin C or colchicine treatments for 3 h followed by a 24 h recovery period. Micronuclei (MN) count for cells cultured in FBS or CDSR-supplemented medium are summarised in Table 1. TK6 cells adapted to CDSR-supplemented medium were in line with cells cultured in FBS-supplemented medium and met successful criteria within OECD TG 487 for MN formation after stimulation with genotoxic compounds.

**TABLE 1:** *In vitro* micronucleus (MN) assay results with mitomycin C (a direct acting clastogen without metabolic activation) and colchicine (an aneugen).

	% MN	% MN
	10% V/V FBS	0% V/V FBS
Untreated (0 μg/mL)	0.2 (±0.4)	0.2 (±0.4)
Mitomycin C (0.05 μg/mL)	1 (±0.8)	1.3 (±0.5)
Mitomycin C (0.15 μg/mL)	2.8 (±3.6)	1.5 (±1.0)
Colchicine (0.03 μg/mL)	11.3 (±5.8)^*^	7.3 (±1.7)^*^
Colchicine (0.09 μg/mL)	7.5 (±2.6)^*^	10.0 (±5.1)^*^
*p < 0.05 (when compared to relevant untreated control)		

## 4 Discussion

Human cells cultured *in vitro* are commonly used to replace *in vivo* animal assessments for toxicity testing, but paradoxically FBS and other animal derived products are still widely used in the culture medium meaning these techniques are not animal-free. These products have two main drawbacks, namely, batch-to-batch variability (making inter- and intra-laboratory reproducibility challenging) and ethical issues. It is well established that batch to batch variability in FBS from different countries of origin and even different lot numbers impacts the characteristics and performance of cells *in vitro* (Stein, 2007; van der Valk et al., 2018). Each serum product is a mixture of proteins, growth factors, electrolytes, lipids, carbohydrates, hormones, enzymes and other miscellaneous undefined molecules (Stein, 2007) and the variability and undefined nature and potential lack of human relevance of these animal-derived products on human cell cultures *in vitro* drives the move to standardised alternatives.

During this study, whilst the FBS used throughout was from a single lot number from the same supplier the inherent variability of cell characteristics over time in culture was captured (Figure 1). While cell viability and cell diameter were constant over time in culture, proliferation rate was more variable, however this inherent variability was considered acceptable as it did not affect cell phenotype and functionality (Figures 5, 6; Table 1). Additionally, inherent variation for TK6 cells in culture parameters has previously been reported in literature, for example, in chromosome alternations (Schwartz et al., 2004).

It is accepted that cell culture media do not create directly comparable conditions with *in vivo* environment, however an awareness of the constituents of the medium and their influence on the cell models can ensure reproducibility of *in vitro* tests and avoid assay interference or incompatibility from components within the media. Therefore, chemically defined serum replacement (CDSR) strategies may be a good solution to improve inherent variability in cell cultures. In CDSR, all the components are identified, alongside their concentrations, which in turn ensures that cells are exposed to the same media composition regardless of batch number, leading to less variability in cell culture parameters. The uptake of CDSR for the production of antibodies and vaccines is well established and more recently within stem cell culture for the potential of cell and gene therapeutics (Schepici et al., 2022). There are already several publications advocating for CDSR application and promoting animal product free research (van der Valk et al., 2010; Gstraunthaler, Lindl and van der Valk, 2013). However, whilst there are several CDSR commercially available the move towards adopting these new media is slow within the *in vitro* cell culture community, mainly due to the time and cost of adapting the cell line and establishing and validating its suitability for use once adapted. Furthermore, the optimisation of medium composition and adaptation protocol is specific for each cell line making this an onerous process.

In this study, we explored two adaptation methodologies i) direct adaptation, where cells were transferred from 10% (v/v) FBS containing medium directly to 0% (v/v) containing medium during one sub-culture (Figure 2) and ii) gradual adaptation, where cells where gradually sub-cultured using culture medium containing reduced concentrations until FBS was completely substituted for CDRS 0% (v/v) (Figure 3). Whilst both methodologies maintained TK6 cell growth in animal-free CDSR medium, the proliferation rate of TK6 cells was significantly reduced using the direct adaptation methodology and hence the gradual adaptation methodology was selected to investigate the long-term effects of CDSR-supplemented medium on TK6 cell properties. Secondly, when cells were successfully adapted (after 14 weeks), they were evaluated in detail to ensure the same characteristics were maintained as displayed in the FBS-containing cultures. TK6 cells did not demonstrate any significant differences outside of the inherent variability of FBS-supplemented TK6 cultures with regards to viability, health or diameter and were maintained within acceptance thresholds typical for FBS-cultured cells. Whilst it is typical to report appropriate cell viability and proliferation characteristics when assessing the suitability of culture medium, few studies are published which confirm cell functionality and cell performance and health over a window of sub-cultures. This study aimed to provide a full evaluation of TK6 cells (including viability, proliferation, morphology, phenotype and functionality) in CDSR-supplemented medium to assess their reproducibility and stability in an animal-product free medium and potential substitute for FBS-supplemented medium in OECD test guidelines.

TK6 are routinely used in OECD TG 487 for detection of genotoxicity. This assessment protocol reports the number of micronuclei formed during cell division and therefore proliferation characteristics (particularly doubling time after subculture) are an important consideration for cell performance when performing the test guideline. In this study, cells cultured in 10% v/v FBS or 10% v/v CDSR had a doubling time of 11.7 and 13.5 h respectively (Figure 4), which was very similar and within the optimal timeframe of 13–15 h reported (Lorge et al., 2016). However, cells cultured in 10% v/v CDSR media had 24 h longer lag phase and the exponential phase of cell proliferation was 48–96 h after subculture. In contrast, cells cultured in 10% v/v FBS displayed exponential growth between 24 and 72 h after cell passaging. A potential explanation for an extended lag phase for CDSR-cultured cells is that gradual adaptation allows cells to adapt to new culture conditions by modifying their gene expression and/or metabolic pathways, utilising alternative sources of growth factors and nutrients for growth (Fang et al., 2017). This difference in lag phase in cell growth does not disqualify cells for the OECD TG 487 assay but it was necessary to take into consideration when performing the assay. Cells cultured in CDSR medium were seeded 24 h earlier for the experiment than cells cultured in 10% (v/v) FBS-containing medium enabling both cultures to reach the exponential phase of cell proliferation prior to initiating the assay. The assessment was performed according to OECD guidelines and both models were exposed to two different micronuclei inducers (mitomycin C and colchicine) in accordance with the short treatment schedule. There was no significant difference (*p* > 0.05) in micronuclei count between cells cultured in FBS-containing or CDSR-containing medium (Table 1).

It has been shown previously that the medium composition may affect cell morphology and can favour certain cell phenotypes or subclones (Tièche et al., 2019; Chary et al., 2022). Therefore, TK6 morphology and phenotype were further characterised by high content image analysis or flow cytometry (Figures 5, 6). Cell morphology is an important aspect of the phenotype of a cell and an important consideration for assessments with morphological endpoints (micronuclei formation). Additionally, changes in morphological parameters as cell size or sphericity may indicate various processes happening in the cell. For example, cell shrinkage, or the loss on cell volume, irregularity in shape, may be a characteristics of a programmed cell death, apoptosis (Bortner and Cidlowski, 2003). In contrast, an increase in cell volume with enlargement of organelles may characterise cell necrosis (Kroemer et al., 2009). TK6 are human lymphoblasts and are small-to -medium in size (approximately 10–18 µm in diameter) with a large nucleus which typically occupies more than 75% of the total cell volume and agranular cytoplasm (Goldsby et al., 1997). In this study, six morphological parameters were assessed: cell and nuclear area, sphericity, number of vacuoles, cell area occupied by vacuoles and total vacuolation area, and no significance difference was observed in comparison to FBS-cultured cells. Moreover, when adapting cells to new conditions there is a risk of selecting a subpopulation of cells with slightly different characteristics, where a minority of the original population can adapt more efficiently to the new conditions. This in turn may cause the cell line to drift towards an alternative phenotype, which may change the cell functionality. To ascertain if the TK6 cell remained as a lymphoblast in CDSR-medium, a phenotypic study of the cells was conducted by flow cytometry. In all cases analysed, nearly 100% of the cells showed CD19 expression and more than 50% of the population showed CD20 expression, as expected from a population of TK6 cells (ECACC Culture Collections). Together, these results demonstrate that new animal-product free cells maintain cell characteristics similar to TK6 cultured in serum containing media.

## 5 Conclusion

This study has demonstrated TK6 cells can be cultured successfully in chemically defined animal product-free media without any significant (*p* > 0.05) effect on cell health, morphology and phenotype both during the adaptation phase and subsequent established long-term culture. To our knowledge, this is the first study of long-term effects of CDSR-supplemented medium on cells. Further studies are needed to determine the suitability of these cells in an extended genotoxicity assessment as well as the viability, functionality and characteristics of cells following freezing process using animal-free cryoprotectants. Although, there are many strategies for cell transition to serum-free medium, results from this study confirm that gradual adaptation is more beneficial for cell health and proliferation. Full characterisation of CDSR-supplemented medium on cell lines is essential to provide the scientific evidence and confidence to move away from FBS and other animal-derived constituents towards CDSR to generate more reproducible and human relevant *in vitro* cell culture assessments.

## Data Availability

The raw data supporting the conclusion of this article will be made available by the authors, without undue reservation.
